# Myostatin Expression, Lymphocyte Population, and Potential Cytokine Production Correlate with Predisposition to High-Fat Diet Induced Obesity in Mice

**DOI:** 10.1371/journal.pone.0012928

**Published:** 2010-09-22

**Authors:** Jeri-Anne Lyons, Jodie S. Haring, Peggy R. Biga

**Affiliations:** 1 Department of Clinical Health Sciences, University of Wisconsin-Milwaukee, Milwaukee, Wisconsin, United States of America; 2 Department of Chemistry and Molecular Biology, North Dakota State University, Fargo, North Dakota, United States of America; 3 Department of Biological Sciences, North Dakota State University, Fargo, North Dakota, United States of America; INRA - Paris 6 - AgroParisTech, France

## Abstract

A strong relationship exists between increased inflammatory cytokines and muscle insulin resistance in obesity. This study focused on identifying a relationship between metabolic propensity and myostatin expression in muscle and spleen cells in response to high-fat diet intake. Using a comparative approach, we analyzed the effects of high-fat diet intake on myostatin and follistatin expression, spleen cell composition, and potential cytokine expression in high-fat diet induced obesity (HFDIO) resistant (SWR/J) and susceptible (C57BL/6) mice models. Results demonstrated overall increased myostatin expression in muscle following high-fat diet intake in HFDIO-susceptible mice, while myostatin expression levels decreased initially in muscle from high-fat diet fed resistant mice. In HFDIO-resistant mice, myostatin expression decreased in spleen, while myostatin increased in spleen tissue from HFDIO-susceptible mice. Proinflammatory cytokine (IL-17, IL-1β, and IFNγ) potential increased in splenocytes from HFDIO-susceptible mice. In comparison, C57BL/6 mice fed a high-fat diet exhibited higher frequencies of CD4^+^/CD44^hi^ and CD8^+^/CD44^hi^ cells in the spleen compared to control fed mice. Together, these results suggest that susceptibility to high-fat diet induced obesity could be influenced by local myostatin activity in a tissue-specific manner and that splenocytes exhibit differential cytokine production in a strain-dependent manner. This study sets the stage for future investigations into the interactions between growth, inflammation, and metabolism.

## Introduction

The pathophysiology of obesity and type 2 diabetes is complex and is associated with environmental and lifestyle risk factors that include a sedentary lifestyle and an overweight condition. The onset of type 2 diabetes can, in most cases, be reversed with lifestyle changes including diet and exercise. However, the mechanisms controlling this pathophysiology are unclear. Gluco- and lipitoxicity are considered to play key roles in the development of type 2 diabetes [1], [2], with the primary molecular defect being impaired insulin-stimulated glucose transport and decreased glycogen synthesis in skeletal muscle [3]. Skeletal muscle represents the most important tissue for maintenance of balanced glucose homeostasis since it accounts for ∼80% of insulin-stimulated glucose uptake [4]. Muscle is also the predominant tissue for whole body lipid oxidation, with up to 90% of the energy requirements at rest being derived from fatty acids [5].

Recently, Zhao et al. [6] demonstrated that disrupting myostatin (MSTN) function by overexpressing its propeptide protects against dietary-induced obesity and insulin resistance. MSTN is a myokine known to negatively regulate muscle growth and impact body fat accumulation. Myostatin is produced as a 375-amino acid propeptide that is proteolytically cleaved at the RSRR (263) site to produce the 26-kDa active MSTN [7]. The resulting prodomain has been shown to inhibit the negative growth activity of active MSTN [8]. Myostatin-null mice show a two-fold increase in muscle mass compared to wild-type mice, as well as a significant suppression of body fat accumulation [9], [10]. Similarly, transgenic mice expressing muscle-specific MSTN propeptide exhibit 20% faster growth and 44% more muscle mass than wild-type controls, but maintain normal adipose tissue [6]. In addition, follistatin has been shown to bind and inhibit MSTN activity [11], [12]. Conversely, overexpression of the active MSTN peptide in muscle results in low muscle weights and increased fat mass [13], suggesting that processing of MSTN may play a role in energy partitioning between protein and fat, counteracting muscle-wasting disorders that are prevalent in many disease states including diabetes.

A strong link exists between increased inflammatory cytokines, such as tumor necrosis factor-alpha (TNF-α) and interleukin-6 (IL-6), and insulin resistance in obese individuals [14]. In response to high fat intake, a low-grade systemic inflammatory response is seen along with increased intramyocellular adipose deposition. A previous study demonstrated that age, intramyocellular lipid, and TNF-α are the strongest predictors of insulin resistance [15]. However, the mechanisms regulating the interactions between the inflammatory response and muscle insulin sensitivity are still not fully elucidated. It has been known for some time that high-fat diets can generally compromise protective immune responses [16], [17]. Recently, the dysfunction of the innate immune response to influenza and increased mortality was linked directly to obesity [18]. It is still unclear where the regulation interactions lie between metabolic and immune response regulation. The intent of the current study was to evaluate the interactions between high-fat diet intake, MSTN expression, and spleen cell population dynamics in two strains of mice exhibiting differential responses to dietary intake. To the authors' knowledge, this is the first report of differential MSTN expression in different strains of mice, as well as the first report of MSTN expression in spleen and leukocytes in mice.

## Materials and Methods

### Ethics Statement

Institutional Animal Care and Use Committees at both University of Wisconsin-Milwaukee and North Dakota State University approved all animal procedures.

### Animals and Experimental Design

Six week old mice, SWR/J and C57BL/6 strains, were used for these experiments (Jackson Laboratories, Bar Harbor, ME). C57BL/6 mice exhibit a high susceptibility to diet-induced obesity, type 2 diabetes, and atherosclerosis [19], [20]. High-fat, high-simple-carbohydrate, low-fiber diet produces obesity in C57BL/6J mice as well as fasting blood glucose levels of greater than 240 mg/dl and blood insulin levels of greater than 150 microU/ml [20], suggesting that the C57BL/6J mouse carries a genetic predisposition to develop non-insulin-dependent (type 2) diabetes. The SWR/J mouse is resistant to diet-induced obesity and does not exhibit glucose intolerance associated with obesity [21]. Mice were allowed to acclimate for a period of two weeks to the facilities, which were maintained at constant 20°C and 50% humidity with a 12∶12 hour light:dark cycle. Mice were acclimated to the control diets for two weeks. Each strain of mouse was divided equally into two experimental groups, which included 1) Control: fed a control diet (10% kcal fat, Research Diets Inc., D12450B), or 2) High-fat: fed a high-fat diet (60% kcal fat, Research Diets Inc., D12492). Mice were fed experimental diets for 12 wk *ad libitum*, and given free access to water. Each treatment group of mice was divided and housed in sub-groups and each experiment was repeated (n = 3/grp, n = 6 total). Individual body weight, whole blood glucose, and average daily food intake were measured weekly. All mice were fasted for 12 h prior to any biological sampling and were euthanized by CO_2_ inhalation prior to end-point sampling (cardiac puncture bleed and tissue collections).

### Sample Collection and Analysis

Tissue and blood samples were collected at end-point sampling for further analyses. Spleen and skeletal muscle (pool of gastrocnemius and soleus) samples were collected and immediately placed on dry ice until further processing. Blood was collected from live animals via the saphenous vein (50 µl) or terminal cardiac puncture (500 µl) for analysis for blood glucose using an Accu-chek Blood Glucose Meter (Roche Diagnostics). Spleen samples isolated at the 6-wk sampling period were used for either RNA isolation (n = 6) or for *ex vivo* lymphocyte activation (n = 4).

### Lymphocyte Activation

T lymphocyte stimulation and activation was performed on individual spleens isolated from SWR and C57BL/6 mice fed either a high-fat or control diet for 6 weeks using modifications to the methods described by Trickett and Kwam [22]. Spleen samples (n = 4) were homogenized by glass dounce homogenizers in Hank's buffered saline to result in a single cell suspension. Following removal of red blood cells and granulocytes using a Percol density gradient, mononuclear leukocytes were suspended in RPMI 1640 medium supplemented with 1% L-glutamine, 1% Hepes, 1% sodium pyruvate, 1% penicillin and streptomycin, and 0.1% beta-mercaptoethanol (complete RPMI, cRPMI). Single cell suspensions were incubated with DynaBeads (CD3/CD28 beads, Invitrogen) to expand and activate T lymphocytes. No difference in total cell counts was detected between treatment groups following cell separation. Cells were plated in cRPMI at a density of 1×10^6^/mL and cultured at 37°C, 10% CO_2_. Aliquots of cell supernatants (100 µL) were collected at 48, 72, and 96 h. Supernatants were spun at 3,000× g for 10 minutes, flash frozen and stored at −80°C until assayed for cytokine production.

### Cellular Composition of Spleen Tissues

Spleens were harvested from all groups of mice into RPMI 1640 supplemented with 10% fetal bovine serum (FBS) and 1% penicillin and streptomycin. Spleens were forced through a mesh screen to obtain a single cell suspension. Red blood cells were removed using RBC Lysis Buffer following manufacturer's directions (eBioscience). Following two washes with supplemented RPMI, splenocytes were counted and aliquots of 1×10^6^ cells were made for each staining combination as well as for flow cytometry compensation controls. Staining was done in labeling buffer (PBS plus 5% FBS). Antibodies used for staining were: FITC anti-mouse CD4, PE-Cy5 anti-mouse CD8, FITC anti-mouse CD19, PE anti-mouse CD44, PE anti-mouse CD25, PE anti-mouse CD69. Cells were stained for 30 minutes at 4°C in the dark. Following one wash in labeling buffer, samples were fixed using Cytofix Solution according to manufacturer's directions (BD), resuspended in 300 µl labeling buffer, and data were collected on a FACSCalibur (BD). Data were analyzed using FlowJo (Treestar).

### Quantitative Real-Time PCR

Changes in genetic expression of myostatin (*MSTN*), follistatin (*FSTN*), and interleukin-6 (*IL-6*) in muscle and spleen samples were evaluated by real-time reverse transcription quantitative PCR (RT-qPCR). Total RNA samples were reverse transcribed using SuperScript II RNase H^-^ reverse transcriptase (Invitrogen) and oligo dT primers to obtain first-strand cDNA. First-strand cDNAs were diluted 1∶20 and used as templates for qPCR analysis. Reactions (25 µl total volume) containing 1 µl diluted template, 150 nM each primer, and 1X Platinum SYBR Green qPCR SuperMix (Invitrogen) were run in triplicate using the Mx3000P real-time PCR system (Stratagene) and the following cycling parameters: 50°C for 5 min; 95°C for 2 min; 40 cycles of 95°C for 15 s, 52°C for 15 s, 72°C for 30 s. No-template controls were run for all primer pairs and PCR efficiencies were calculated for each primer pair. Mean cycle threshold values (Ct values) for each target gene were normalized with mean beta-actin Ct values. Primer PCR efficiencies were calculated and utilized for PCR correction for all primer pairs and normalized data were analyzed using the standard curve quantification method [23].

### Myostatin Protein Production Analysis

Total muscle and spleen MSTN protein levels were detected using Western ligand blot techniques. Total tissue proteins (10 µg) were separated on reducing SDS-PAGE gels (8–16% Tris-HCl, Pierce) and transferred to nitrocellulose membranes (ProTran, 0.45 µm). Myostatin prodomain immunoreactive peptides (MPIPs) were detected with anti-GDF8/myostatin prodomain antibody (AF-1539, R&D Systems). Putative pro-myostatin (∼50 kDa) and prodomain (∼37 kDa) were detected in muscle, while putative processed prodomain (∼32 and 35 kDa) and further processed immunoreactive peptides (∼15 kDa) were detected in spleen tissue.

### Cytokine Expression Analysis

Cytokine expression was evaluated in cultured, activated spleen lymphocyte populations utilizing a commercially available protein array that recognizes 12 inflammation-related cytokines, including granulocyte macrophage-colony stimulating factor (GM-CSF), interferon-γ (IFNγ), IL-1α, IL-1β, IL-2, IL-4, IL-5, IL-6, IL-10, IL-12, IL-17, and TNFα. The ExcelArray™ Mouse Inflammation Array (82005, ThermoFisher) was utilized to detect the expression of cytokines by activated splenic lymphocytes isolated from high fat fed and control animals of both strains of mice. The array is a multiplex fluorescent sandwich ELISA that contains internal normalization, standards for quantification, and is optimized for cell culture supernatants. The assay was performed according to the recommendations of the manufacturer. Briefly, neat cell culture supernatants were incubated on the slides for 2 h, and a streptavidin detection system was utilized to visualize. Visualization was conducted two ways to ensure accuracy of detection. Slides were sent to the manufacturer for slide reading and data acquisition, while other slides were scanned, analyzed, and data acquired using the GenePix Array Scanner and software. No differences were detected between data sets utilizing the two scanning techniques.

### Statistical Analysis

All RT-qPCR data were analyzed using the standard curve method where cycle threshold values were compared to standard curves generated and validated for each primer pair to result in a relative starting copy number of mRNA. All comparisons within strains were analyzed by two-way analysis of variance (ANOVA) with factors being time and treatment using GraphPad Prism version 5.0c for Mac OS X, GraphPad Software, San Diego California USA, www.graphpad.com. Bonferroni post-hoc comparisons were conducted when overall interactions were significant (P<0.05) to test for differences between treatment groups at each sampling time.

All cytokine, weight, and blood glucose data were analyzed using three-way analysis of variance (StatPlus, AnalystSoft) with factors being strain, time, and treatment. Pairwise multiple-comparison tests were conducted by the Holm-Sidak or Tukey-Kramer method to compare between and within strains only when main effects were significant. Cell frequencies were analyzed using two-way analysis of variance with factors being strain and time. Pairwise comparisons were conducted as previously stated. Normality of sample distribution was tested by Normal Quantile Plot (Q-Q Plot), and residuals were plotted against predicted values to evaluate dependency between the means and variances and test the assumption of homogeneity of variances. Results were reported as least square means ± SE.

## Results

### SWR mice are resistant to high-fat diet induced obesity

**Figure 1:**
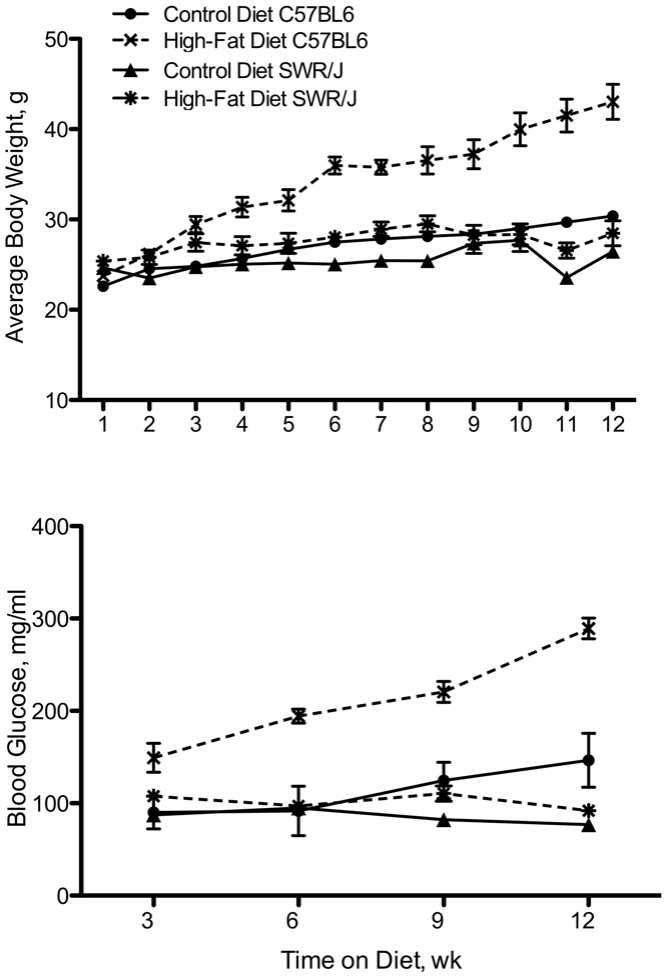
SWR/J mice are resistant to HFDIO. Body weight (a) and whole blood glucose (b) in C57BL/6 and SWR/J mice strains fed experimental diets for 12 wk. Control diet  = 10% kcal fat, high-fat diet  = 60% kcal fat.

Over the 12-wk feeding trial, C57BL/6 mice fed the high-fat diet consistently gained weight, while the other treatment groups failed to exhibit significant weight gain (Fig. 1a). Significant weight increases were detected as early as 3 weeks on the high-fat diet in C57BL/6 mice. At 12 wk, the high-fat fed C57BL/6 mice nearly doubled their weight from wk 1, 43.03±0.10 g vs. 24.25±0.08 g. No difference was detected between the control- and high-fat fed SWR mice throughout the trial. In addition, there was no difference between SWR mice (control or high-fat) and the control C57BL/6 group at any time point in the feeding trial.

Consistent with weight changes between the experimental groups, the C57BL/6 high-fat fed group exhibited increased blood glucose levels at 3, 6, 9, and 12 wk following dietary intake (Fig. 1b). Overall, blood glucose levels were elevated 2.45-fold in C57BL/6 mice fed the high-fat diet compared to mice fed the control diet (236±50 mg/dl compared to 96±10 mg/dl). However, SWR mice fed a high-fat diet did not exhibit elevated blood glucose levels compared to control group (94±7 mg/dl vs. 81±14 mg/dl). These levels were significantly lower than the C57BL/6 high-fat group, but not different from the C57BL/6 mice fed the control diet. In addition, there was no difference detected in voluntary feed intake between any group of mice throughout the feeding trial (data not shown).

### High-fat diet induced obesity results in differential molecular regulation of growth-related genes in muscle and spleen tissue

**Figure 2:**
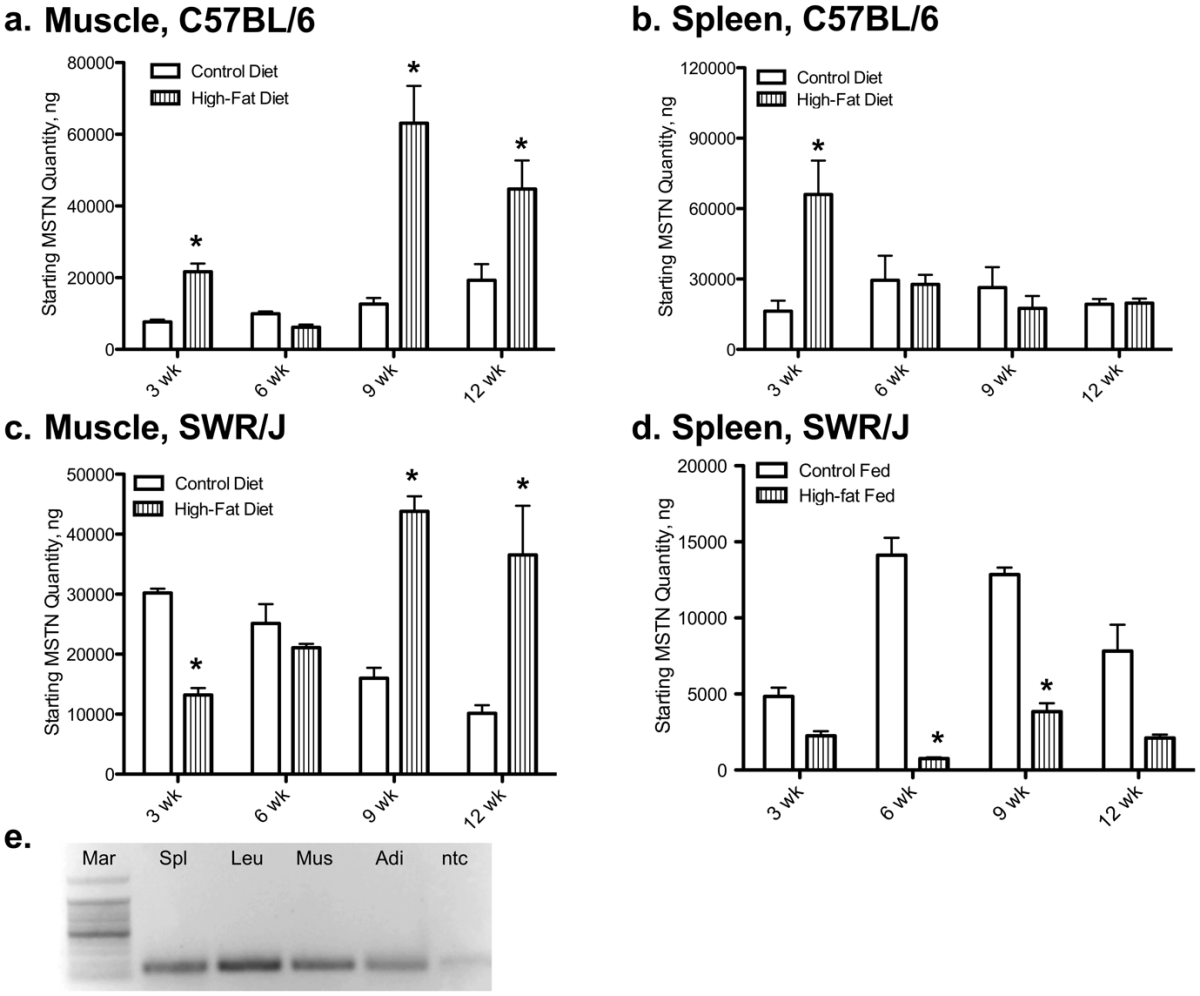
Myostatin is attenuated in spleens of HFDIO-resistant mice fed high fat. Molecular regulation of *myostatin* (*MSTN*) mRNA levels using quantitative real-time PCR. *Myostatin* mRNA levels in muscle from C57BL/6 (a) and SWR/J (c) and spleen from C57BL/6 (b) and SWR/J (d). Muscle and spleen samples were taken at 3, 6, 9, and 12 wk on the dietary treatment. (e) RT-PCR analysis of *myostatin* mRNA expression in different tissues for verification. Mar) 100 bp ladder, Spl) whole spleen, Leu) isolated leukocytes (CD3/CD28), mus) pooled skeletal muscle, Adi) adipose tissue, and ntc) no template control. * demonstrates between dietary treatment differences at a given time, P<0.05.

No difference was detected in *IL-6* mRNA levels in muscle or spleen of C57BL/6 or SWR mice fed a high-fat diet. In addition, no differences were detected between *IL-6* mRNA levels in C57BL/6 or SWR control fed mice in either tissue. In contrast, *MSTN* mRNA levels were increased by high-fat diet intake in muscle tissue of SWR and C57BL/6 mice after 9 and 12 wk, respectively (Fig. 2a and 2c). At 3 wk, *MSTN* mRNA levels were decreased in SWR muscle while levels increased in C57BL/6 muscle. Levels of *MSTN* mRNA were higher at 3 and 6 wk, and lower at 12 wk, in muscle from control SWR mice compared to control C57BL/6 mice. SWR spleen *MSTN* mRNA levels decreased at 6 wk and 9 wk on the high-fat diet (Fig. 2d). C57BL/6 spleen *MSTN* levels increased 8.84±0.13-fold at 3 wk on the high-fat diet, with no change detected at 6, 9, or 12 wk (Fig. 2b). *MSTN* mRNA levels in spleen of SWR mice fed the high-fat diet were consistently lower than control fed mice, regardless of strain. No difference was detected in *MSTN* mRNA levels between C57BL/6 or SWR control mice. The presence of *myostatin* in whole spleen and isolated leukocytes was confirmed through traditional RT-PCR (Fig. 2e).

**Figure 3:**
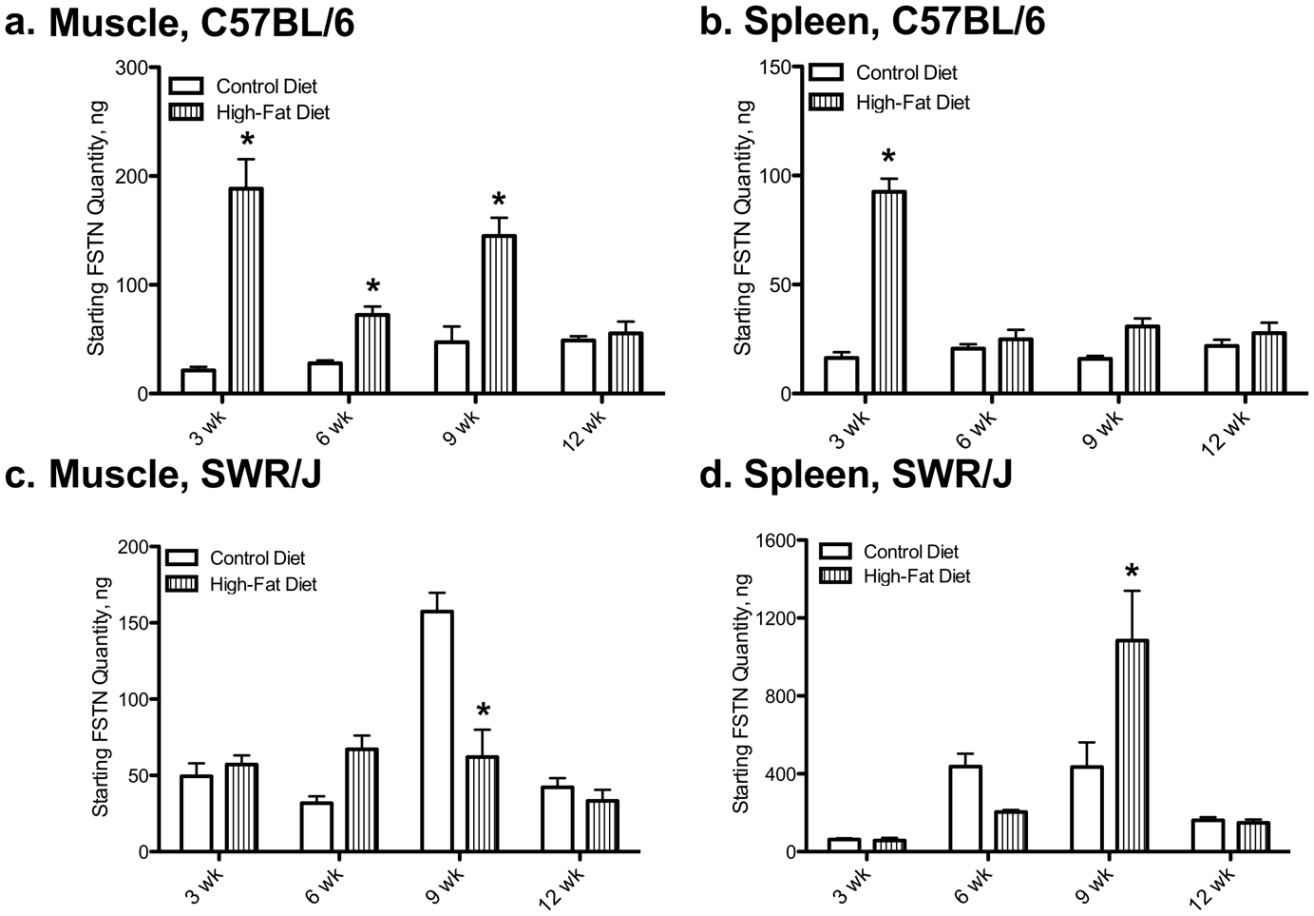
Follistatin is increased in muscle of HFDIO-susceptible mice fed a high-fat diet. The molecular regulation of *follistatin* (*FSTN*) mRNA levels was analyzed by real-time PCR. *Follistatin* mRNA levels in muscle from C57BL/6 (a) and SWR/J (c) and spleen from C57BL/6 (b) and SWR/J (d). Muscle and spleen samples were taken at 3, 6, 9, and 12 wk on the dietary treatment. * demonstrates between dietary treatment differences at a given time, P<0.05.

*Follistatin* levels were similar to *MSTN* levels in muscle tissue of C57BL/6 mice, with an increase (Fig. 3a) at 3, 9, and 12 wk. However, *FSTN* mRNA levels in muscle of high-fat fed SWR mice were lower than control fed levels at 9 wk (Fig. 3c). Control FSTN levels were higher in SWR muscle than C57BL/6 muscle only at the 9 wk sampling period. In C57BL/6 mice, *FSTN* mRNA levels in spleen increased following 3 wk feeding of high-fat diet compared to control fed animals (92.50±8.36 compared to 16.34±2.35 copies, respectively; Fig. 3b). Similarly in SWR high-fat fed mice, spleen *FSTN* mRNA levels increased compared to control fed levels (1084±46.56 compared to 434.6±18.67), but not until 9 wk on the diet (Fig. 3d). Control spleen levels of *FSTN* mRNA were higher in SWR mice at both 6 and 9 wk.

### Unprocessed myostatin levels are higher in susceptible mice than resistant mice

**Figure 4:**
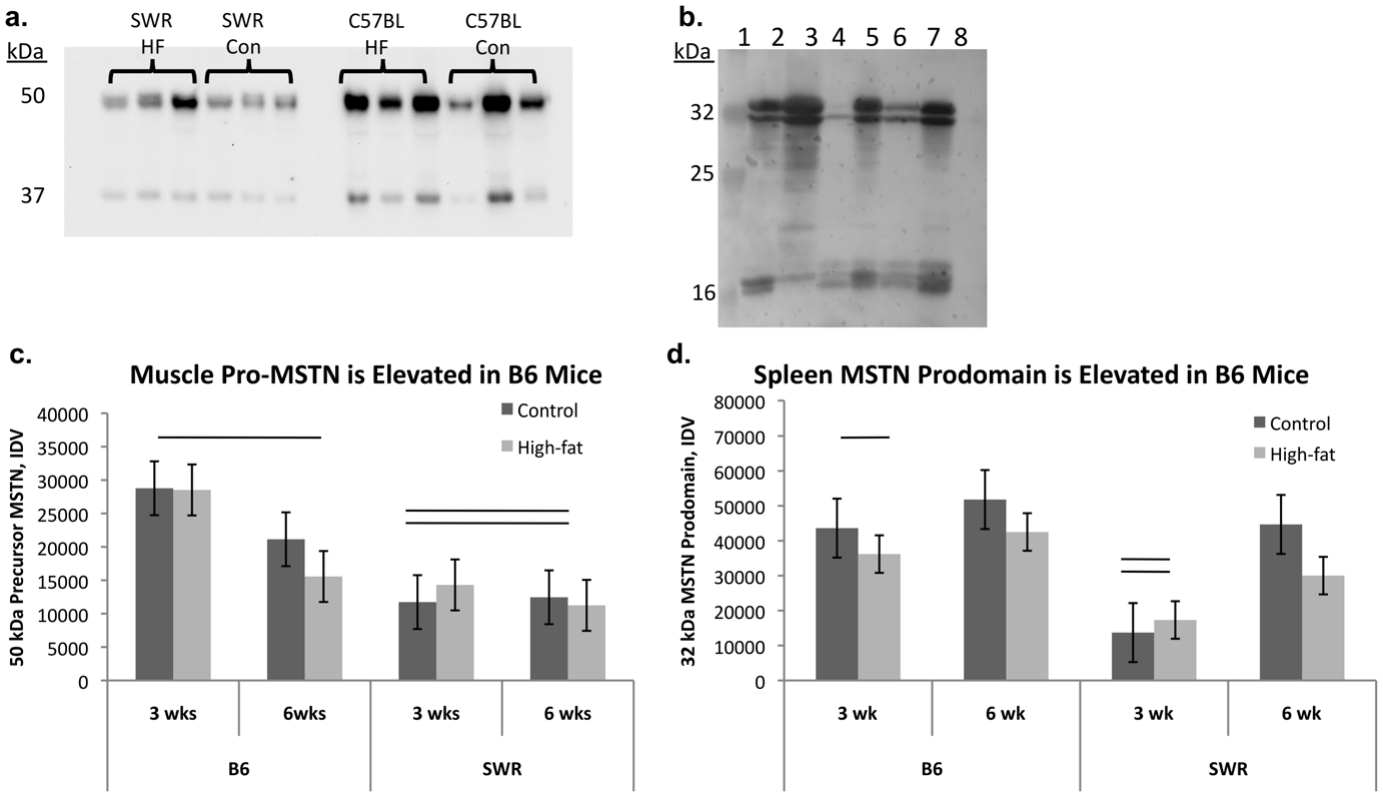
Precursor myostatin expression is higher in HFDIO-susceptible mice. Representative Western ligand blots demonstrating total muscle (a) and spleen (b) myostatin prodomain immunoreactive peptides (MPIPs). In muscle tissue, MPIPs were detected at 37 and 50 kDa, representing the prodomain of myostatin and the precursor protein, respectively. In spleen tissue, MPIPs were detected at 16 and 32 kDa, representing processed prodomain peptides and potentially further processed peptides, respectively. a.) Representative muscle protein samples from SWR/J and C57BL/J high-fat (HF) and control [7] fed mice. b.) Representative spleen protein samples. Lane 1: molecular weight marker, 2,3,5,7: C57BL/6 spleen, 4,6: SWR spleen, 8: -control, SWR adipose tissue protein. c, d. Relative quantification of arbitrary densitometry values from C57BL/6 (B6) and SWR mice fed control or high-fat diets for 12 weeks. No differences were detected at 9 or 12 wks for any MPIP or for muscle 37 kDa, spleen 16 kDa MPIPs, data not shown. Differences marked with lines were considered significantly different at *P*<0.05.

Myostatin prodomain immunoreactive peptides were detected in both muscle and spleen tissue from C57BL/6 and SWR mice (Fig. 4a, b). Myostatin immunoreactive peptides of ∼50 and 37 kDa, consistent with precursor MSTN and MSTN prodomain respectively, were detected in muscle tissue from both C57BL/6 and SWR mice (Fig. 4a). No differences were detected in MSTN prodomain expression levels between strains, diets, or over time on the diets (Fig. 4c). Precursor MSTN expression levels were higher in muscle from C57BL/6 mice compared to SWR regardless of diet (Fig. 4c). Myostatin immunoreactive peptides of ∼32 and 16 kDa, consistent with processed MSTN prodomain and potentially further processed MSTN prodomain respectively, were detected in spleen tissue protein from both C57BL/6 and SWR mice (Fig. 4b). Two MSTN immunoreactive peptides were detected between 30 and 33 kDa. Both MPIPs (32 and 31 kDa) were expressed at higher levels in C57BL/6 spleen than in SWR spleen regardless of treatment (Fig. 4d). No differences were detected between strains or as an effect of diet on the 16 kDa MPIP.

### Cytokine production potential is affected by both high-fat diet intake and strain

**Figure 5:**
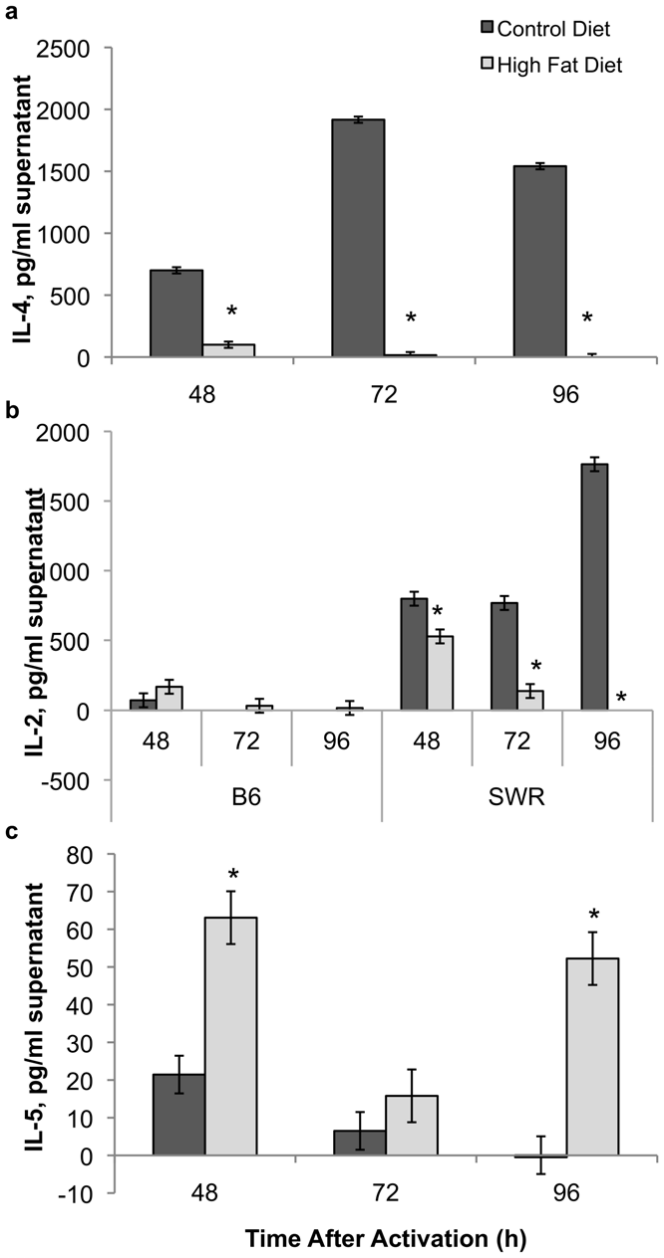
IL-4 levels are attenuated in HFDIO-resistant mice when fed a high fat diet. Cytokine levels in *ex vivo* CD3^+^/CD28^+^ splenocytes from SWR and C57BL/6 (B6) mice at 48, 72, and 96 h after activation. IL-4 (a), IL-2 (b), and IL-5 (c) levels were measured by cytokine expression array. IL-4 and IL-5 levels were not detectable in C57BL/6 mice splenocytes in this assay. * P<0.05.

IL-4 expression by activated CD3/CD28 splenic T cells isolated from SWR mice was consistently decreased in high-fat fed mice compared to control (e.g., low-fat fed) mice at all sampling times (Fig. 5a). Similarly, IL-2 levels were decreased in SWR mice on the high-fat diet compared to the control diet at all time points after lymphocyte activation (Fig. 5b). In C57BL/6 mice, the high-fat dietary treatment resulted in an increase in IL-2 levels by spleen cells at 48 h T cell activation with levels almost twice as high as those in control mice. IL-2 levels were consistently higher in the high-fat fed C57BL/6 group compared to the low-fat fed group, with levels below the detection limit in the latter group at 72 and 96 h. This is in direct contrast to SWR mice, with consistently lower IL-2 levels expressed by high-fat fed mice than controls. IL-5 levels were elevated in the high-fat fed SWR group compared to control fed mice (Fig. 5c). In contrast, both IL-4 and IL-5 levels were below the detection limit in spleen cell supernatants from C57BL/6 mice at all sampling times and treatments groups.

**Figure 6:**
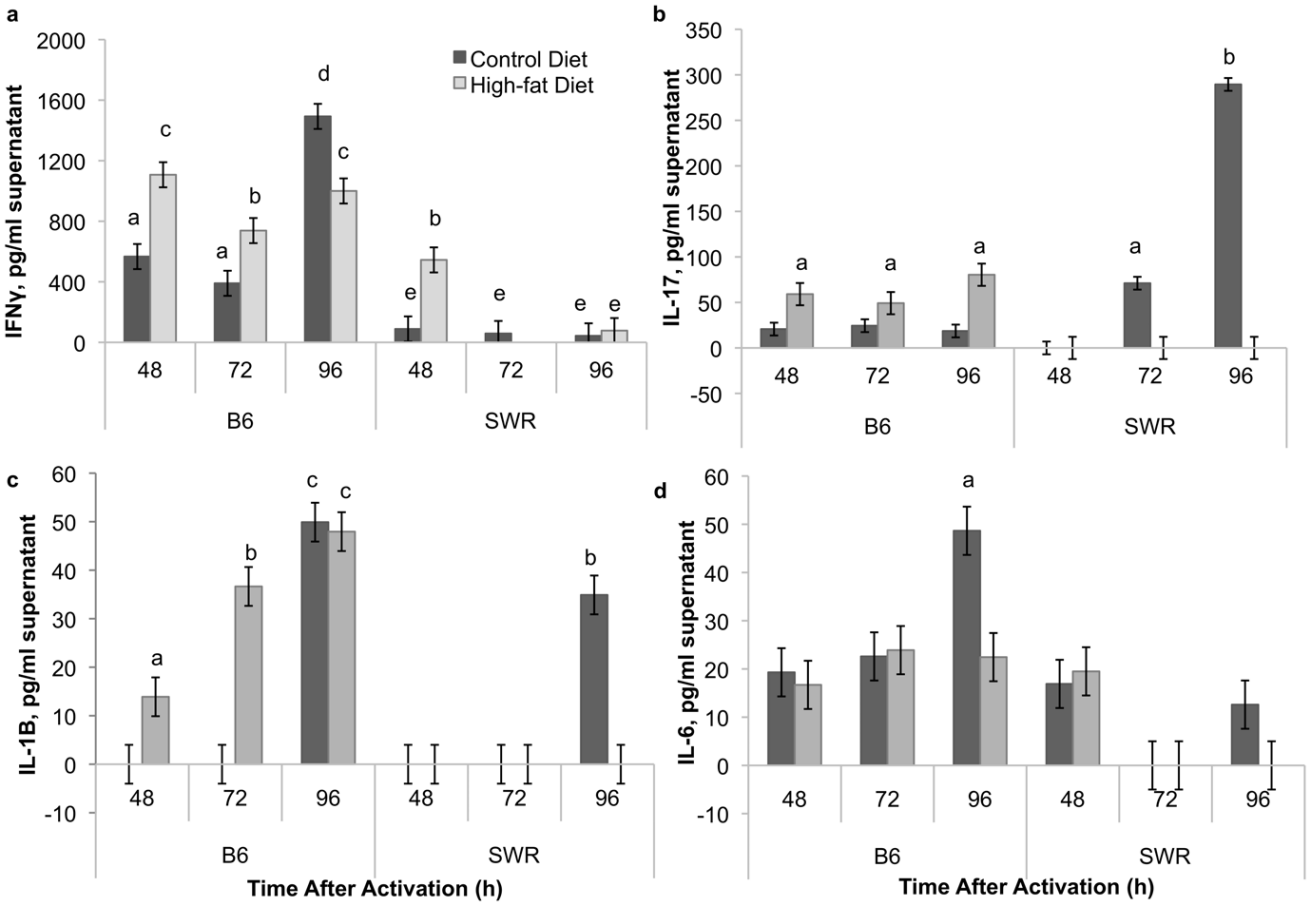
Pro-inflammatory cytokines are highest in HFDIO-susceptible mice. Cytokine expression from CD3^+^/CD28^+^ splenocytes from C57BL/6 (B6) and SWR mice 48, 72, and 96 h after activation. IFNγ (a), IL-17 (b), IL-1β (c), and IL-6 (d) protein levels. Different letters represent significance at P<0.05.

Interferon-γ (IFNγ) levels were consistently higher in C57BL/6 mice, regardless of dietary treatment, compared to SWR mice, with the exception of the high-fat fed SWR group at 48 h after activation (Fig. 6a). IL-17 levels were elevated in C57BL/6 high-fat fed mice compared to controls at all time points (Fig. 6b). In comparison, IL-17 levels were undetectable at 48 h post activation in both control and high-fat fed groups in SWR mice. Levels increased at 72 and 96 h in control compared to high-fat fed SWR mice spleen lymphocytes. IL-1β levels were elevated in high-fat fed C57BL/6 spleen cell supernatants at 48 and 72 h (Fig. 6c), while levels in C57BL/6 control mice were undetectable. At 96 h, no difference was detected between control and high-fat C57BL/6 mice. IL-1β levels in SWR spleen supernatants were undetectable, with the exception of control sample at 96 h. IL-6 levels were consistently detected in C57BL/6 spleen lymphocytes from both treatment groups, but only detected at 48 h in both treatments in SWR mice spleen cell supernatants (Fig. 6d). IL-6 levels were elevated in control C57BL/6 mice at 96 h compared to high-fat fed mice.

**Figure 7:**
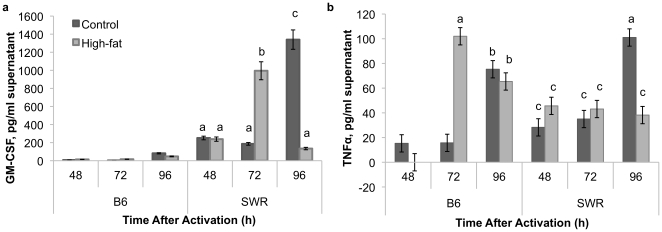
GM-CSF levels are higher in HFDIO-resistant mice. Granulocyte macrophage colony stimulating factor (GM-CSF) (a) and tumor necrosis factor alpha (TNFα) (b) levels in activated CD3^+^/CD28^+^ splenocytes from C57BL/6 (B6) and SWR mice fed control or high-fat diets for 12 weeks. Different letters represent significance at P<0.05.

Granulocyte macrophage colony stimulating factor (GM-CSF) levels were elevated in SWR mice compared to C57BL/6 at all time points (Fig. 7a). No difference was detected between control and high-fat fed mice at 48 h in SWR mice, but at 72 h GM-CSF levels were elevated in SWR high-fat fed mice spleen lymphocyte supernatants compared to control. At 96 h, GM-CSF levels were elevated in SWR control spleen lymphocytes compared to high-fat SWR. Differences were not detected in tumor necrosis factor-α (TNFα) levels between treatment groups in SWR mice at 48 or 72 h, but levels were elevated at 96 h in control spleen samples (Fig. 7b). In C57BL/6 mice, TNFα levels were elevated at 72 h in high-fat fed mice compared to controls, and no differences were detected at 48 or 96 h. Levels of IL-1A, IL-10, and IL-12 were below detection limits of the assay (12.0 pg/ml).

### Differences in lymphocyte populations were detected between susceptible versus resistant strains of mice following flow cytometric analysis of splenic lymphocyte populations

**Figure 8:**
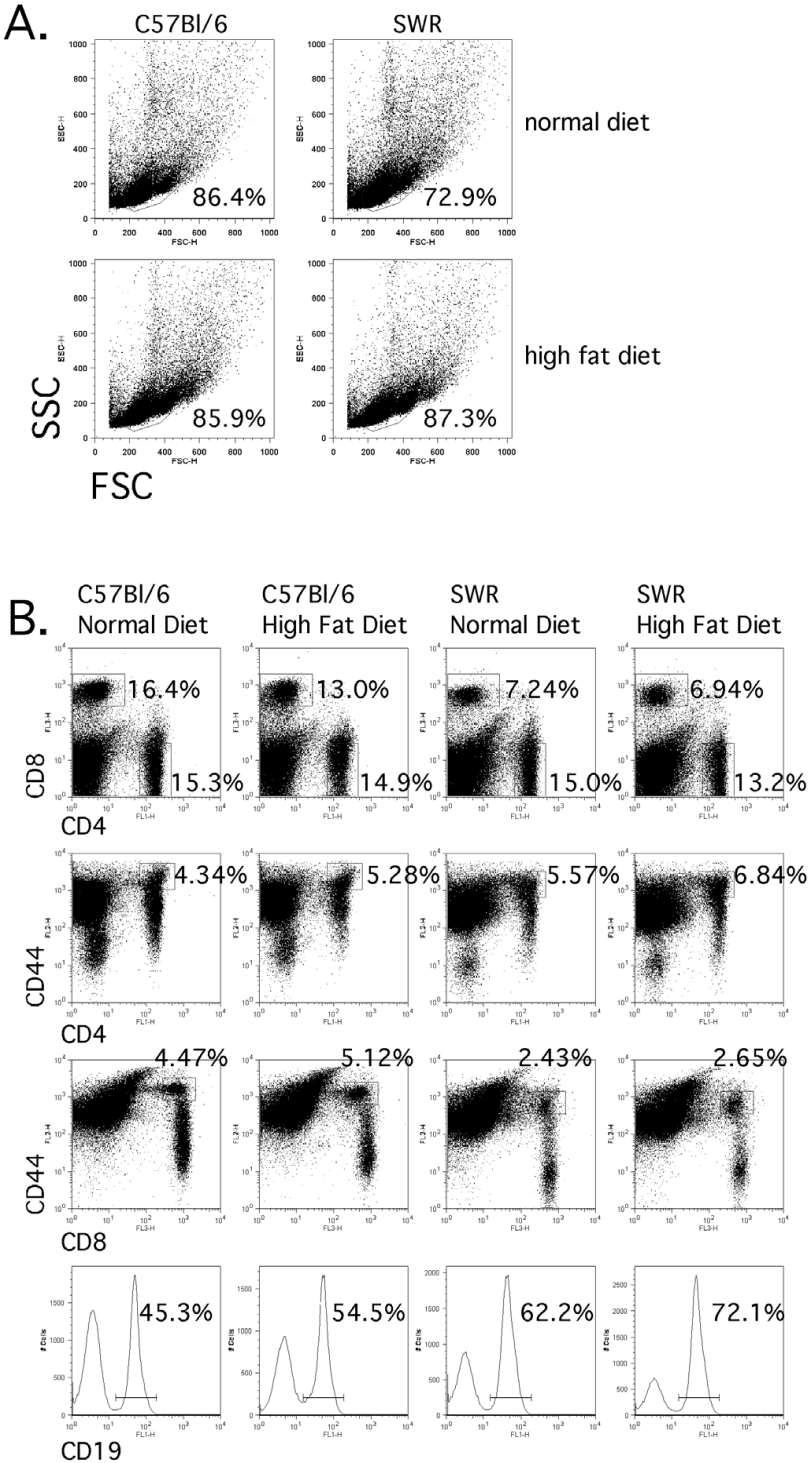
T cell population frequency percentages were not different between strains. Splenocytes from identically housed C57BL/6 and SWR/J mice fed control or high-fat diet were isolated and cell frequencies were measured by flow cytometry. A) Total lymphocyte frequencies by strain and diet. B) Frequencies of CD4^+^, CD8^+^, CD4^+^/CD44^hi^, CD8^+^/CD44^hi^, and CD19 cells were determined.

**Figure 9:**
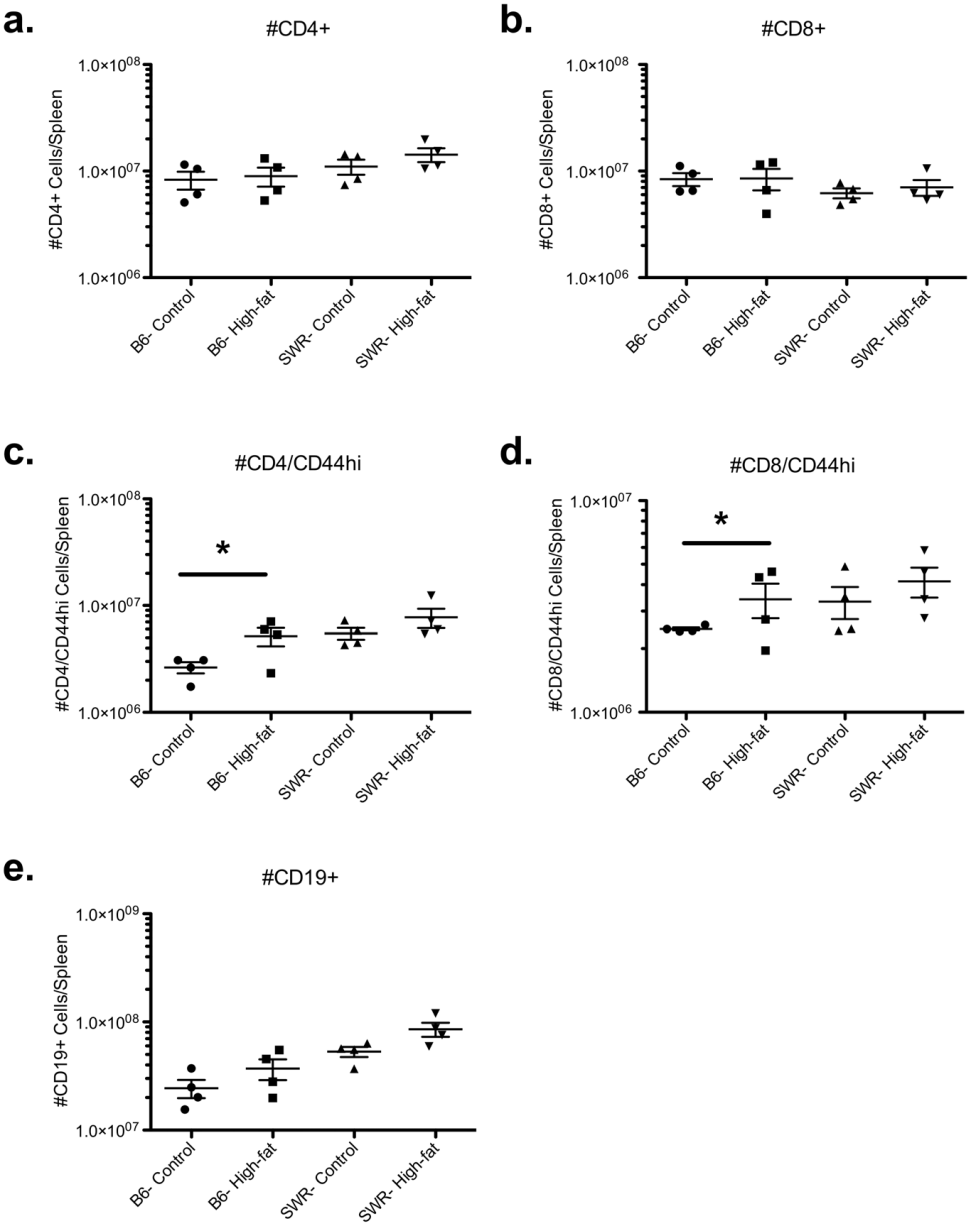
Higher frequencies of previously activated CD4^+^ and CD8^+^ T cell populations observed in HFDIO-susceptible mice. To address strain- or diet-specific shifts in lymphocyte populations, an evaluation of spleen cell frequencies of CD4^+^ (a) and CD8^+^ (b) T cells, CD19^+^ B cells (e) was conducted in naive C57BL/6 (B6) and SWR/J (SWR) mice fed control or high-fat diets. Cell populations were also stained for previously activation marker CD44^hi^, CD4/CD44^hi+^ (c) and CD8/CD44^hi+^ (d). Data are presented as total positive lymphocyte numbers/spleen. The total number of cells counted was not significantly different between strains or dietary treatment. * represents differences between pairwise groups (P<0.05, n = 4).

Measurement of inflammatory cytokine production following global activation of splenic T cells from susceptible and resistant mice fed either control or high-fat diets revealed distinct differences, which suggests that the lymphocytic environment is quite different both before and after the institution of dietary treatment. Splenocytes harvested from identically housed groups of mice were analyzed for differences in lymphocyte frequencies both to investigate possible sources of these inflammatory cytokines, and to determine potential downstream effects these differential cytokine potentials may exact on lymphocyte population dynamics. Frequencies of CD4^+^, CD8^+^, and CD19^+^ cells were determined as well as the frequency of cells expressing CD25 or CD69, which would be indicative of recent activation, and CD44, which is a cumulative measure of past activation (Fig. 8). No significant differences in total CD4^+^, CD8^+^, or CD19^+^ lymphocyte populations were detected between C57BL/6 and SWR mice regardless of dietary treatment (Fig. 9). However, within C57BL/6 mice the frequency of previously activated CD8^+^ and CD4^+^ T cells (CD8^+^/CD44^hi^ and CD4^+^/CD44^hi^) were elevated in high-fat fed mice compared to controls (Fig. 9).

## Discussion

### C57BL/6 mice are highly susceptible to high-fat diet induced obesity, while SWR mice are resistant

As anticipated, C57BL/6 mice are highly susceptible to high-fat diet induced obesity, with a significant increase in weight seen as early as three weeks on the diet. Following 12 wk on a high-fat diet, C57BL/6 mice almost doubled their initial weight. Consistent with these results, a similar increase in whole blood glucose levels was detected in C57BL/6 mice on the high-fat diet. In addition, glucose levels continue to rise in high-fat fed C57BL/6 mice as time on the diet increases. In contrast, there were no differences detected in weight or whole blood glucose levels in SWR mice over the 12-week feeding trial. These data suggest that the SWR strain of mice is resistant to the body weight and insulin challenges seen in C57BL/6 mice on a high-fat diet and is consistent with previous work [24], [25].

**Figure 10:**
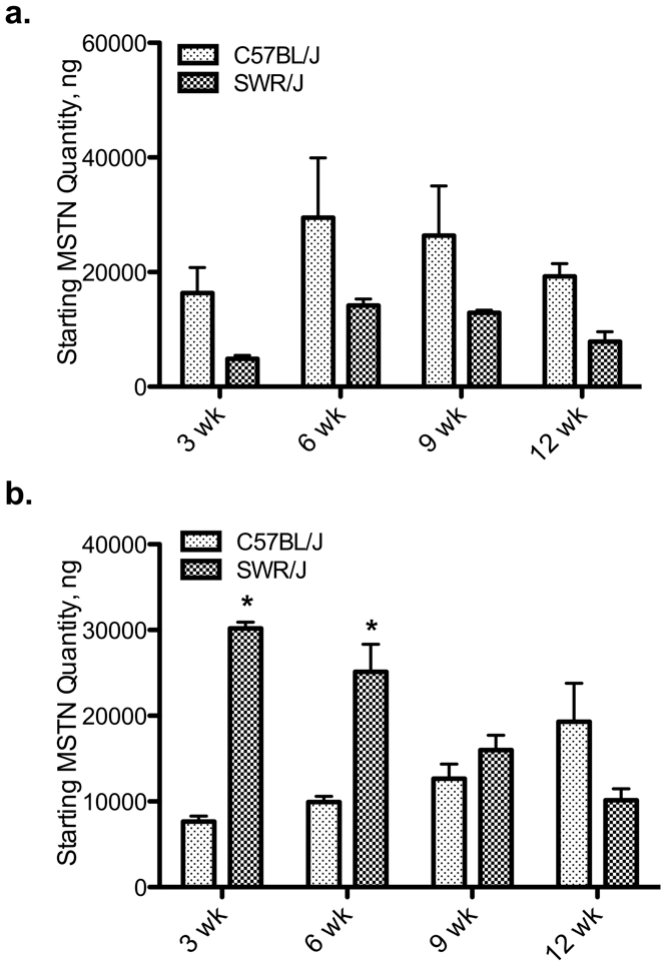
Muscle myostatin levels in control fed animals were higher in HFDIO-resistant mice. *Myostatin* mRNA levels from control fed C57BL/6 and SWR/J mice at 3, 6, 9, and 12 weeks of the experiment from spleen (a) and muscle (b) tissue. * represents differences between pairwise groups, P<0.05.

This is the first report of differential *MSTN* regulation in a non-muscle tissue, and specifically expression in isolated leukocytes from a mammalian species (Fig. 2e). As expected, *MSTN* levels were significantly different between strains in response to high-fat diet intake. Most notable is the dramatic decrease in *MSTN* levels in whole spleen tissue of SWR mice in response to a high-fat diet. Overall levels of *MSTN* mRNA in control SWR spleen appear lower than levels detected in C57BL/6 spleen tissue but were not significantly lower (Fig. 10a), possibly due to the high variability detected in C57BL/6 control spleen *MSTN* mRNA levels. However, in SWR mice fed a high-fat diet *MSTN* mRNA levels were consistently lower than levels observed in control SWR or C57BL/6 mice, suggesting a different regulatory mechanism is activated in response to high-fat diet intake in HFDIO-resistant animals. A dramatic increase in spleen *MSTN* mRNA levels was detected in C57BL/6 mice with 3 wk of high-fat diet intake and no differences detected at other sampling times. These results are intriguing as they are consistent with previous reports of MSTN knockout mice being resistant to high-fat diet induced obesity [26], [27] as HFDIO-resistant mice in this study exhibit lowered levels of *MSTN* expression. However, one difference is that our study demonstrates a dramatic down-regulation of *MSTN* in spleen tissue, with only an initial decrease in *MSTN* levels in muscle tissue following 3 wk of high-fat diet intake. Guo and co-workers demonstrated that removal of myostatin activity in adipose tissue results in no differences to body composition, weight gain, glucose or insulin sensitivity, and were not resistant to HFDIO, suggesting that metabolic changes in skeletal muscle are required for improved HFDIO-resistance and metabolic handling.

In muscle tissue, *MSTN* mRNA levels were elevated in C57BL/6 in response to high-fat diet, as well as in SWR mice following 9 and 12 wk of high-fat diet intake. Interestingly, control levels of *MSTN* were higher in muscle from SWR mice compared to control C57BL/6 mice (Fig. 10b). It is possible that higher levels of *MSTN* mRNA in younger SWR mice play an important role in sensitizing them to metabolic shifts compared to C57BL/6 mice. It is possible that timing of high-fat diet intake affects the animal's ability to handle the excess lipid. In older SWR mice, around 20 weeks of age (respective to the 12 wk sampling time), muscle *MSTN* levels are reduced compared to C57BL/6 levels. Myostatin has been shown to promote adipogenesis *in vitro* [28], [29], and in this study we demonstrate that in HFDIO-resistant mice increases in lipid consumption initially decrease *MSTN* levels in muscle and spleen tissue with more of an effect in spleen tissue. Taken together with Guo and co-worker's results demonstrating that removal of MSTN activity in muscle is required for HFDIO resistance, we suggest that MSTN might play a substantial role in metabolic shift regulation that occurs in muscle cells during increased lipid intake that results in either increased glucose uptake or increases intramyocellular lipid accumulation, depending on resistance of susceptibility. It is also likely that myostatin plays a significant role in spleen tissue as well.

Different sized immunoreactive peptides were detected between the two tissues, indicating potential tissue-specific processing of MSTN. Results indicate that only strain has an effect on MSTN protein levels and that dietary lipid intake does not have any effect on MSTN protein processing. In C57BL/6 muscle tissue, precursor MSTN (∼50 kDa) expression levels were higher than SWR mice with no effect of diet. In addition, processed MSTN prodomain levels were elevated in C57BL/6 spleen tissue compared to SWR regardless of dietary treatment. These differences in strain expression of MSTN could help explain relative differences in sensitivity to glucose uptake and adipogenesis between the strains. However, the exact regulatory mechanisms of MSTN action are not clear *in vivo*, and recent evidence indicates that MSTN activity might be sequestered when it is bound to components of the extracellular matrix [30], [31], [32]. This level of activity control makes it difficult to interpret changes, or lack there of, in steady-state expression of MSTN. However, to further support the effect of high-fat diet intake on *MSTN* levels, changes in *follistatin* mRNA levels were similar to *MSTN* levels with consistent upregulation observed in C57BL/6 in response to lipid intake. Collectively, these results indicate that MSTN plays a role in HFDIO susceptibility and resistance and that tissue-specific expression likely contributes to the role MSTN plays in metabolic regulation.

Differences in *IL-6* expression levels were not different between dietary treatment groups in muscle or spleen tissue from C57BL/6 or SWR mice. This is interesting because IL-6 levels are known to increase in circulation in obese and diabetic states [33], [34], and IL-6 is known to regulate glucose homeostasis [35], [36]. The lack of mRNA expression level differences between the strains of mice suggests that either IL-6 production increases are a result of adipose IL-6 production, translation rate increases, or post-transcription modification changes. In addition, not all obese/diabetic states are IL-6-dependent.

### Cytokines are Responsive to Dietary Intake and Strain-Specific Expression

Low-grade inflammation commonly observed with obesity is thought to play an important role in increasing risk for metabolic disorder [37], [38]. Tissue inflammation following high-fat intake results from the recruitment and activation of local macrophages that release inflammatory cytokines that are thought to promote insulin resistance locally [37], [38], [39]. Most attention has been paid to adipose, skeletal muscle, and liver tissue in this regard. In this project we focus on the changes in cytokine expression potential in spleen tissue due to the dramatic differences observed in myostatin expression between HFDIO-resistant and susceptible mice. Cytokine expression data supports interactions between strain, diet, and potential immune system regulation. IL-6 is thought to play an important role in the inflammatory-related pathogenesis of obesity. Interestingly, no difference was detected in *IL-6* levels in total spleen or muscle from either mouse strain. However, IL-6 protein expression by lymphocytes isolated from low-fat-fed C57BL/6 mice was elevated following 96 h of activation by CD3/CD28 stimulation, which results in activation of the total T lymphocyte population. Interestingly, levels dropped below the detection limit of the assay in SWR mice 72 and 96 h following activation in both feeding groups, suggesting a strain-dependent difference in potential activation response.

IL-4 is a cytokine (prototypically Th2) important for B cell activation, antibody production, Th2 cell proliferation and differentiation, as well as in macrophage fusion and myoblast recruitment and fusion [40], [41]. IL-4 is also important in down-regulating the production of IFNγ, a key proinflammatory cytokine. IL-4 expression was consistently lower in high-fat fed SWR activated lymphocytes compared to the control fed SWR group at all sampling times, while expression was undetectable in C57BL/6 mice at all time-points tested, regardless of diet. Interestingly, a recent report demonstrated that central IL-4 administration in conjunction with a high-fat diet led to hypothalamic inflammation and HFDIO [42], indicating a pro-inflammatory response to IL-4. It is likely that IL-4 functions as pro- or anti-inflammatory in response to the cellular and physiological environment.

IL-2 expression potential was similar to that of IL-4, as IL-2 levels decreased in high-fat diet fed SWR mice compared to control diet fed mice at all time points. IL-2 is a cytokine known to maintain T regulatory cells for self/non-self recognition during infection, as well as stimulate growth, differentiation, and survival of T cells. IL-5, important in stimulating B cell growth, levels were elevated in SWR mice compared to C57BL/6 mice regardless of diet. In contrast to IL-4, IL-5 expression was higher in high-fat fed SWR mice compared to control fed animals. Together the data reported here indicate that cytokine expression potential of splenoctyes may play particularly important roles in the ability of SWR mice to handle an increased lipid load without increasing weight or lipid deposition or any apparent pro-inflammatory response.

In contrast to the expression of anti-inflammatory cytokines in response to high-fat diet in the obesity-resistant SWR animal, obesity-prone C57BL/6 animals presented with evidence of an increased inflammatory response resulting from the high-fat diet. When compared to SWR mice, IFNγ levels were consistently higher in activated T lymphocytes from C57BL/6 mice spleen regardless of dietary treatment, with the exception of the high-fat fed SWR group at 48 h after activation. IFNγ is a hallmark Th1-type cytokine that, among its many roles, stimulates inflammation. Macrophages accumulate in adipose tissue during obesity [43] and IFNγ produced by these macrophages was recently shown to regulate fat inflammation during obesity [44]. In addition, evidence suggests that the increase in systemic TNF-α in obesity is derived from these adipose macrophages, which suggests that this increase in infiltrated macrophages might represent the cause or consequence of the low-grade inflammation observed in obesity [45], [46], [47], [48]. The increased potential of IFNγ production by C57BL/6 activated splenic lymphocytes and potentially macrophages, presented here is consistent to the previously described systemic inflammation regulation.

The most notable differences reported are the strain difference in response to high-fat diet intake on IL-17 and IL-1β levels, both cytokines that, along with IFNγ, can drive the inflammatory response. In C57BL/6 mice, high-fat diet increased both cytokines expression level potential with a time-dependant increase in IL-1β levels. Levels of IL-17 and IL-1β were not detectable in high-fat fed SWR mice, and only detectable in control mice over time. The increased IL-1β, IL-17, and IFNγ potential levels detected in C57BL/6 mice fed a high-fat diet were not correlated (*P* = 0.85), however a clear strain difference is detected. Thus, the splenic environment of HFDIO-susceptible (C57BL/6) mice could contribute to increased systemic inflammation seen in respond to a high-fat diet, compared to the more protective anti-inflammatory response that is possible in SWR mice.

Lymphocyte populations present in the spleens of SWR and C57BL/6 mice in both dietary treatment groups were similar with respect to CD4^+^ and CD8^+^ T cell groups, but higher frequencies of both previously activated CD4^+^ and CD8^+^ cells (CD44^hi^) were detected in C57BL/6 mice fed a high-fat diet. Interestingly, CD8^+^/CD44^hi^ T cells have previously been identified as a potent source of IFN-γ [49], [50]. The fact that C57BL/6 mice have a greater frequency of these cells compared to SWR mice suggests that they may be a potential source for the increased IFN-γ detected in the cytokine analysis.

A proinflammatory phenotype is associated with obesity and insulin resistance [37], [38]. Data presented here suggested a genetic link between diet, myostatin expression, and potential for generating a proinflammatory response. Consistent with previous reports of obesity-prone C57BL/6 mice responding to a high-fat diet with the induction of a proinflammatory response [37], [38], this work demonstrates increased spleen production potential of IFNγ, IL1β, and IL-17. In contrast, obesity-resistant SWR mice exhibited an increased spleen potential for lymphocytes to produce greater levels of IL-4 and depressed potential for production of proinflammatory cytokines.

The results of this study clearly show an interesting link between susceptibility to high-fat diet induced obesity and myostatin expression. Myostatin has been shown to regulate adipogenesis and cell proliferation [29], [51] and be regulated by stress [52], [53], [54]. It is clear that several factors confound the predisposition to HFDIO, however it is apparent that myostatin plays a role in regulating cell cycle regulation and metabolism and might be acting as a sort of regulatory switch for metabolic shifts in a tissue-specific manner. In HFDIO-resistant mice, myostatin levels are consistently lower in spleen tissue than in susceptible mice. This evidence provides further support that myostatin and its activity are potential therapeutic targets for metabolic regulation. It is possible that myostatin also serves as a modulator for innate immune system regulation involved in inflammation progression as dynamic changes were detected in splenocytes following high-fat dietary intake. Further investigations are needed to empirically identify regulatory interactions between metabolic- and inflammatory-regulating pathways to better understand the propensity for diabesity and to provide opportunities for prevention.
